# Impact of Chatbot-Based Cognitive Training on Cognitive Function and White Matter Integrity in Cognitively Unimpaired Older Adults: Randomized Controlled Trial

**DOI:** 10.2196/94127

**Published:** 2026-10-28

**Authors:** Bori R Kim, Eunhee Kim, Soo Mee Lim, Jinwoo Kim, Jee Hyang Jeong, Geon Ha Kim

**Affiliations:** 1 Department of Neurology College of Medicine, Ewha Womans University Mokdong Hospital Ewha Womans University Seoul Republic of Korea; 2 Ewha Medical Research Institute, Ewha Womans University Seoul Republic of Korea; 3 Department of Radiology College of Medicine, Ewha Womans University Mokdong Hospital Ewha Womans University Seoul Republic of Korea; 4 Department of Neurology College of Medicine, Ewha Womans University Seoul Hospital Ewha Womans University Seoul Republic of Korea; 5 Department of Digital Therapeutics Research and Development HAII Corp. Seoul Republic of Korea; 6 Graduate School of Cognitive Science Yonsei University Seoul Republic of Korea

**Keywords:** aging, chatbot, cognitive training, diffusion tensor imaging, randomized controlled trial, white matter

## Abstract

**Background:**

Cognitive training may enhance cognition and support brain health during aging, but few trials have evaluated readily accessible chatbot-based programs with neuroimaging outcomes.

**Objective:**

This study aimed to evaluate the effects of a chatbot-based cognitive training program on white matter microstructural organization and cognition in cognitively unimpaired older adults.

**Methods:**

In a single-blind randomized controlled trial, 72 cognitively unimpaired older adults were assigned to chatbot-based cognitive training (30 min/day, 5 days/week for 12 weeks; n=37) or a no-intervention control (n=35). Diffusion tensor imaging at baseline and 12 weeks quantified fractional anisotropy (FA) in predefined white matter tracts, and cognition was assessed with the Cambridge Neuropsychological Test Automated Battery.

**Results:**

Groups were comparable at baseline. After 12 weeks, the training group showed significantly less FA reduction than controls in the bilateral tapetum and right posterior thalamic radiation, surviving false discovery rate control (*P*<.05). The training group also improved on Paired Associates Learning (PAL), an episodic memory measure (*P*=.04). PAL improvement was positively associated with FA change in the right posterior thalamic radiation and right sagittal stratum (false discovery rate–adjusted *P*=.04 for each).

**Conclusions:**

These findings suggest that a 12-week chatbot-based cognitive training program may support episodic memory and the relative maintenance of white matter microstructural organization in selected pathways in cognitively unimpaired older adults, consistent with a brain-maintenance framework.

**Trial Registration:**

CRIS KCT0004630; https://tinyurl.com/4xfesb68

## Introduction

Extensive research indicates that cognitive training can mitigate age-related cognitive decline, with improvements reported across multiple domains including memory, attention, and executive functions [1-3]. Large-scale randomized controlled trials, such as the Advanced Cognitive Training for Independent and Vital Elderly (ACTIVE) trial, have demonstrated domain-specific gains in memory, processing speed, and reasoning, with benefits extending to everyday functioning over long-term follow-up [4,5]. However, many traditional interventions rely on in-person group delivery, which may be less accessible for older adults due to mobility or transportation barriers, scheduling constraints, or reluctance to participate in group-based settings. In addition, such programs often require trained facilitators, limiting scalability across communities [6]. These limitations underscore the need for accessible, scalable, home-based cognitive interventions, particularly when in-person delivery is constrained [7].

Computerized (digital) cognitive training offers a scalable alternative that can be delivered at home with fewer logistical barriers [8]. Particularly, chatbot-based cognitive training represents an evolution of mobile-delivered computerized cognitive training. By integrating conversational AI, chatbots simulate human-like interactions that provide social-cognitive stimulation, which is often lacking in passive digital platforms. This interactive nature may improve engagement and adherence—key challenges in home-based interventions for older adults—by reducing perceived technological burden and providing interactive guidance and feedback [9,10]. This is especially important for older adults living alone, where home‑based assistive technologies can maintain accessibility without spatial or scheduling limitations [11,12]. Early studies suggest that chatbot interfaces reduce technological burden while supporting key adherence factors such as usability and consistent feedback [8,13].

Building on prior evidence, we developed Saemi, a chatbot-based cognitive training program delivered through KakaoTalk, a mobile messaging platform widely used in Korea [14,15]. Saemi provides structured cognitive training tasks targeting memory, attention, calculation, language, and executive function. Each daily session includes at least 2 different training modules: one consistently focused on memory and the other targeting another cognitive domain. This design emphasizes memory training as a recurring component, which is consistent with the evidence that memory decline is a key early feature of age-related cognitive changes [16-18]. By leveraging the interactive nature of chatbots, the program enhances personalization and user engagement, while its home-based format helps overcome spatial and logistical barriers, ultimately promoting sustained cognitive stimulation through accessible mobile technology.

While chatbot-based interventions represent a promising and scalable approach, their cognitive benefits and potential neurobiological correlates remain insufficiently characterized in cognitively unimpaired (CU) older adults. Relative maintenance of brain structural integrity—particularly in white matter (WM)—is considered a key mechanism supporting cognitive resilience during aging and is central to the brain maintenance framework [19-21]. WM integrity, particularly in tracts facilitating interhemispheric communication and sensory-cognitive integration, is essential for efficient neural communication and is strongly associated with memory, attention, and executive function [22-24]. Such WM microstructural changes may be sensitive to aging and experience, and may reflect plasticity-related processes over time [25-27].

Therefore, this study aimed to determine whether a 12-week chatbot-based cognitive training program could attenuate age-related decline in WM integrity and concurrently enhance cognitive performance, consistent with a brain maintenance framework. By addressing both neural mechanisms and cognitive outcomes, this work sought to evaluate the potential of a scalable, home-based intervention to support brain maintenance and cognitive resilience in aging.

## Methods

### Study Design

This was a prospective, single-center, rater-blind, randomized controlled trial, which was conducted from November 21, 2019, to July 13, 2020, at the Memory Disorder Clinic of Ewha Womans University Mokdong Hospital in Seoul, Republic of Korea. This study was reported in accordance with the CONSORT-EHEALTH (Consolidated Standards of Reporting Trials of Electronic and Mobile Health Applications and Online Telehealth) checklist (Multimedia Appendix 1). Patients and the public were not involved in the design, conduct, reporting, or dissemination plans of this trial.

### Participants

A total of 81 CU community-dwelling individuals were recruited from the Ewha Womans University Mokdong Hospital in Seoul. All participants underwent a comprehensive neuropsychological assessment using the Seoul Neuropsychological Screening Battery, second edition (SNSB-II) [28] by trained clinical neuropsychologists. CU was defined as those aged 60 or above who scored more than –1 SD below the mean of age- and education-matched norms in tests evaluating memory, attention, language, visuospatial, and frontal executive functions in the SNSB-II. We excluded individuals with any of the following characteristics: (1) suspected or confirmed mild cognitive impairment or dementia; (2) suspected or confirmed major neurological or psychiatric illnesses, including major depressive disorders; (3) contraindications to MRI; (4) visual or hearing impairments severe enough to interfere with questionnaire response; (5) history of medications that could affect cognitive and emotional functions in the past 3 months; and (6) other major medical problems. Of the 81 participants, nine were excluded because of the withdrawal of consent before the baseline assessment. Therefore, a total of 72 participants were included in this study.

### Sample Size

For the formal sample size calculation, we used the cortical thickness effect-size estimate specified in the study protocol: an expected longitudinal cortical thickness change of 0.046 with an SD of 0.053, corresponding to a standardized effect size of Cohen *d*=0.87 [29]. The sample size calculation was based on a 2-tailed independent-samples *t* test comparing mean longitudinal change between the 2 groups. Assuming 2 parallel groups, a 2-tailed α of .05, and 90% statistical power to detect group differences, the required sample size was estimated to be 30 participants per group. To accommodate an anticipated dropout rate of approximately 20%—commonly observed in MRI-based intervention studies—we planned to enroll 36 participants per group, yielding a total target sample of 72 participants.

### Ethical Considerations

Written informed consent was obtained from all participants. The study protocol was approved by the Institutional Review Board (IRB) of Ewha Womans University Mokdong Hospital (IRB number 2019-06-028). The study was conducted in accordance with the Declaration of Helsinki and relevant guidelines and regulations. Participant data were handled confidentially and deidentified for analysis in accordance with applicable institutional policies. Participants did not receive financial compensation for participation. This trial was registered with the Clinical Research Information Service (CRIS), Republic of Korea (KCT0004630).

### Randomization

After the baseline assessment, 72 participants were randomly allocated in a 1:1 ratio to 2 groups: the intervention group (n=37) and the control group (n=35). A simple, computer-generated randomization sequence was created by an independent statistician who had no contact with study participants. To ensure allocation concealment, the sequence was stored in a password-protected file and revealed only after the baseline assessment was finalized. Trained research staff enrolled participants and had no access to the allocation sequence. Outcome assessors were blinded to group assignments throughout the study.

### Blindness

All outcome evaluators were blinded to the group assignment of participants and were not involved in the intervention.

### Intervention

All participants from both groups received 30 minutes of education about dementia prevention after undergoing the baseline assessment. Participants in the control group received education only and were instructed to continue their usual activities for 12 weeks without any additional cognitive training. This education included the definition, diagnosis, and current treatments of dementia and lifestyle modifications for dementia prevention, encompassing the importance of cognitive training, healthy foods for the brain, aerobic exercise, and stretching that could be done at home [30,31]. Beyond the study intervention, no other cointerventions were provided. To avoid confounding effects, participants continued their routine health care and agreed not to participate in any other cognitive enhancement programs throughout the trial.

All participants in the intervention group used Saemi (HAII Corporation), a chatbot-based cognitive training program delivered via a mobile messaging platform. The program provides representative brain-training tasks grouped by cognitive domains—language, memory, attention, calculation, and executive function—as illustrated in Figure 1. Participants were instructed to complete training for at least 30 minutes per day, 5 days per week, for 12 weeks. Each daily session comprised at least 2 tasks, including one consistently focused on memory and another targeting a different domain (language, visuospatial function, calculation, or executive function). Actual intervention exposure was quantified using app usage logs. Usage metrics included total training time, completed session counts, active days, and mean use time per active day. Time-based adherence rate was calculated as the percentage of the recommended minimum total training time completed according to app usage logs. The prescribed minimum dose was 30 minutes per day on 5 days per week for 12 weeks, corresponding to 30 total hours across the intervention period. Adverse events were monitored throughout the trial via participant self-reports and spontaneous reporting.

The figure shows example tasks targeting language, memory, attention, calculation, and executive function in a chat-based mobile format.

**Figure 1:**
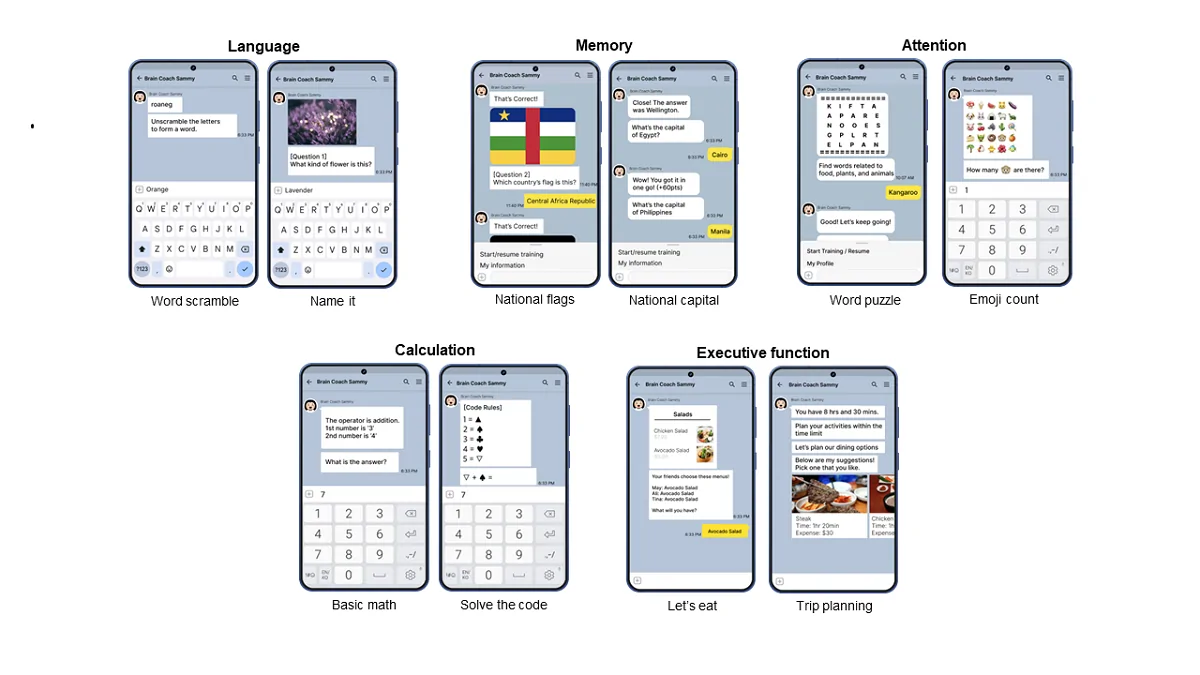
Representative interfaces of the Saemi chatbot-based cognitive training program across cognitive domains.

### Outcome Measures

#### Changes in WM Microstructure

WM microstructural changes were assessed using diffusion tensor imaging (DTI). Fractional anisotropy (FA) was the principal WM metric due to its sensitivity to microstructural organization and its association with cognitive performance in aging [22,32]. Longitudinal FA changes were evaluated using predefined tract-level region-of-interest (ROI) measures extracted from tract-based spatial statistics (TBSS)–processed skeletonized diffusion maps, rather than as a single global FA average; therefore, false discovery rate (FDR) correction was applied across the predefined ROIs. Global baseline FA was used only to assess baseline comparability between groups. Additional metrics such as mean diffusivity (MD), axial diffusivity (AD), and radial diffusivity (RD) were analyzed as complementary measures to provide a broader characterization of WM changes. In line with conventional neuroimaging approaches in intervention studies, WM changes were evaluated as longitudinal alterations over the intervention period, with FA used as the principal imaging marker.

#### Cognitive Outcomes

Episodic memory performance was assessed using the Paired Associates Learning (PAL) task of the CANTAB [33,34]. PAL was selected because memory training was the core component of the intervention, and PAL is highly sensitive to early episodic memory decline and intervention-related improvements in CU older adults [35]. Raw scores were standardized to *z* scores based on the baseline mean and SD of the control group, with positive *z* scores reflecting better performance. PAL was the principal cognitive measure of episodic memory, reflecting its established validity as a sensitive measure of associative episodic memory.

In addition to PAL, 5 further tasks were included to assess additional cognitive domains: Delayed Matching to Sample (DMS), Rapid Visual Information Processing (RVP), Reaction Time (RTI), Spatial Working Memory (SWM), and One Touch Stockings of Cambridge (OTS). Raw scores were standardized to *z* scores as for PAL, with positive *z* scores representing better performance [36]. The models for cognitive outcomes were adjusted for age, sex, years of education, and baseline CANTAB scores; however, the reported *P* values were not corrected for multiple comparisons across cognitive outcomes.

### MRI Acquisition and Processing

#### MRI Acquisition

All participants underwent brain MRI on the same scanner (Achieva, Philips 3.0T) using a standardized imaging protocol. The 3D T1 turbo field echo magnetic resonance images were acquired with the following imaging parameters: sagittal slice thickness=0.5 mm; no gap; repetition time (TR)=9.9 ms; echo time (TE)=4.6 ms; flip angle=8°; and a matrix size of 240×240 pixels. Fluid-attenuated inversion recovery magnetic resonance images were used to evaluate abnormal WM hyperintensities. These were assessed using the following imaging parameters: axial slice thickness=2 mm; no gap; TR=11000 ms; TE=125 ms; inversion time=2800 ms; matrix size=256×189 pixels. For TBSS, DTI were acquired using a diffusion-weighted single-shot echo-planar imaging sequence with the following parameters: TR=9.2-9.9 s; TE=91 ms; flip angle=90°; b-value=1000 s/mm^2^; matrix size=128×126 pixels; 70 axial sections; slice thickness=2 mm; in-plane resolution=1.72×1.72 mm. With the baseline image without diffusion weighting (the reference volume), diffusion-weighted images were acquired from 32 different directions.

#### DTI Processing

DTI data were processed using the Functional Magnetic Resonance Imaging of the Brain (FMRIB) Software Library (FSL, v6.0.2). Because the JHU ICBM-DTI-81 white-matter labels atlas has been reported to contain left-right orientation and label inconsistencies in some distributed versions [37], we performed an explicit left-right orientation check after registration to standard space and verified atlas orientation before ROI masking and extraction [38,39]. Eddy current–induced distortions and head motion were corrected by affine registration of each diffusion-weighted volume to the non–diffusion-weighted (b0) image using eddy_correct (FSL), which relies on the FMRIB’s Linear Image Registration Tool (FLIRT) for linear registration [40,41]. The b vectors were then rotated appropriately using the “fdt-rotate-bvecs” tool as part of FSL [38,42]. Diffusion tensors were then calculated at the level of an individual voxel to generate FA images using DTIfit, and the images of brain tissue were subsequently extracted using the Brain Extraction Tool [43] from the FSL.

The baseline and follow-up FA data of all subjects were realigned onto the standard FMRIB58_FA template provided by the FSL software using a nonlinear registration algorithm implemented in the TBSS package. To improve anatomical longitudinal alignment, we used FLIRT tools from FSL [40,41]. The registered FA images were averaged to generate a skeletonized mean FA image, which represents the main fiber tracts and the center of all tracts common to the group. A threshold of 0.2 was applied to exclude peripheral tracts where there was significant intersubject variability and/or partial volume effect due to gray matter. Each participant’s aligned FA data was projected onto the mean skeleton to create a skeletonized FA map. A similar process was performed for the MD, AD, and RD. The skeletonized diffusion maps were subsequently used for atlas-based ROI extraction and ROI-level statistical analyses.

#### ROI Selection

Following TBSS-based preprocessing and skeletonization, ROI analyses were performed to obtain anatomically interpretable tract-level diffusion measures. ROIs were defined using the JHU ICBM-DTI-81 white-matter labels atlas (48 tracts) [44,45]. The final 17 ROIs were selected based on prior neuroanatomical and cognitive-aging literature, rather than on Shapley additive explanations (SHAP) results. As a supplementary exploratory analysis, SHAP feature-importance ranking across all 48 atlas-defined regions was examined in relation to PAL change (Figure S2 in Multimedia Appendix 2) and was not used to define the ROI set, to support confirmatory inference, or as a criterion for statistical significance [46]. The selected ROIs included the splenium of the corpus callosum, bilateral tapetum, bilateral anterior corona radiata (ACR), bilateral posterior thalamic radiation (PTR; including optic radiation), bilateral sagittal stratum (SS), bilateral cingulum (cingulate gyrus and hippocampal segments), bilateral superior longitudinal fasciculus (SLF), and bilateral uncinate fasciculus. The 17 selected ROIs and their corresponding JHU ICBM-DTI-81 atlas label numbers are provided in Table S3 in Multimedia Appendix 2. ROI-level diffusion metrics were extracted for each selected tract and used as longitudinal outcome measures.

### Statistical Analyses

Statistical analyses were performed using STATA software (version 18; StataCorp). Baseline demographic and clinical characteristics were compared between groups using the chi-square test for categorical variables, independent-samples *t* tests for continuous variables, and the Mann-Whitney *U* test for nonnormally distributed variables. Baseline neuropsychological test performances were compared using analysis of covariance adjusted for age, sex, and years of education.

For cognitive outcomes, raw scores from each task were transformed into standardized *z* scores using the mean and SD of the control group at baseline; where necessary, scores were oriented such that higher *z* scores indicated better performance [47].

The main analytical approach for longitudinal changes in DTI metrics and CANTAB outcomes was a linear mixed-effects model, including fixed effects for group, visit (baseline vs follow-up), and the group-by-visit interaction, with a participant-specific random intercept to account for within-subject correlations. Models were adjusted for age, sex, years of education, and the baseline value of each outcome. Missing outcome data were handled implicitly using maximum likelihood estimation in the mixed-effects framework; inference relies on the standard missing-at-random assumption [48].

The main analysis followed the intention-to-treat (ITT) principle, including all randomized participants in the groups to which they were assigned, regardless of adherence. In addition, a supplementary per-protocol (PP) analysis included only those participants who completed the intervention and the follow-up assessment [49].

ROI diffusion metrics were analyzed using the same mixed-effects framework. To account for multiple comparisons across ROIs, FDR correction was applied, with Bonferroni-adjusted and uncorrected *P* values reported for descriptive purposes.

Associations between changes in WM microstructure (DTI metrics) and cognitive performance (CANTAB *z* scores) were examined using partial correlation analyses, adjusting for age, sex, years of education, and baseline imaging and cognitive scores. For these exploratory structure-function analyses, missing values were handled using multiple imputation by chained equations with predictive mean matching (m=20 imputations; 5 iterations) under a missing-at-random assumption, implemented in R (version 4.4.1; *mice* package) [50]. Imputed datasets were analyzed separately and combined using standard multiple-imputation pooling rules [51]. Imputation diagnostics were evaluated using density plots. *P* values were adjusted for multiple comparisons using the Benjamini-Hochberg FDR procedure.

### Protocol Deviations

This trial was prospectively registered with the CRIS of the Republic of Korea (KCT0004630), with cortical thickness and resting-state functional magnetic resonance imaging (fMRI) specified as the registered primary imaging outcomes. These modalities were acquired as planned; however, modality-specific longitudinal analyses of cortical thickness and resting-state fMRI have not yet been completed and are not included in the present report. DTI was included from the outset in the IRB-approved neuroimaging protocol but was not separately listed as an outcome in the CRIS registration. Because DTI processing and longitudinal analyses had been completed for the cohort, this study focuses on longitudinal changes in DTI-derived WM microstructure together with cognitive outcomes and should be interpreted as a focused analysis rather than as a report of the registered primary imaging outcomes. Separately, the registered protocol described a 60-minute daily cognitive training schedule, whereas the implemented intervention instructed participants to complete at least 30 minutes per day, 5 days per week for 12 weeks. This change was made before participant enrollment to reduce participant burden and improve the feasibility of sustained home-based training in older adults.

## Results

### Baseline Characteristics

Of the 72 participants who underwent baseline assessment, 9 (12.5%) participants withdrew from the study: 5 (13.5%) from the intervention group and 4 (11.4%) from the control group. Consequently, 63 (87.5%) participants completed the final assessment (Figure 2). No intervention-related adverse events were reported in either group. Baseline demographic characteristics, clinical variables, neuroimaging characteristics, and cognitive performance across attention, visuospatial function, memory, language, and frontal executive domains did not differ significantly between the intervention and control groups (Table 1).

**Figure 2:**
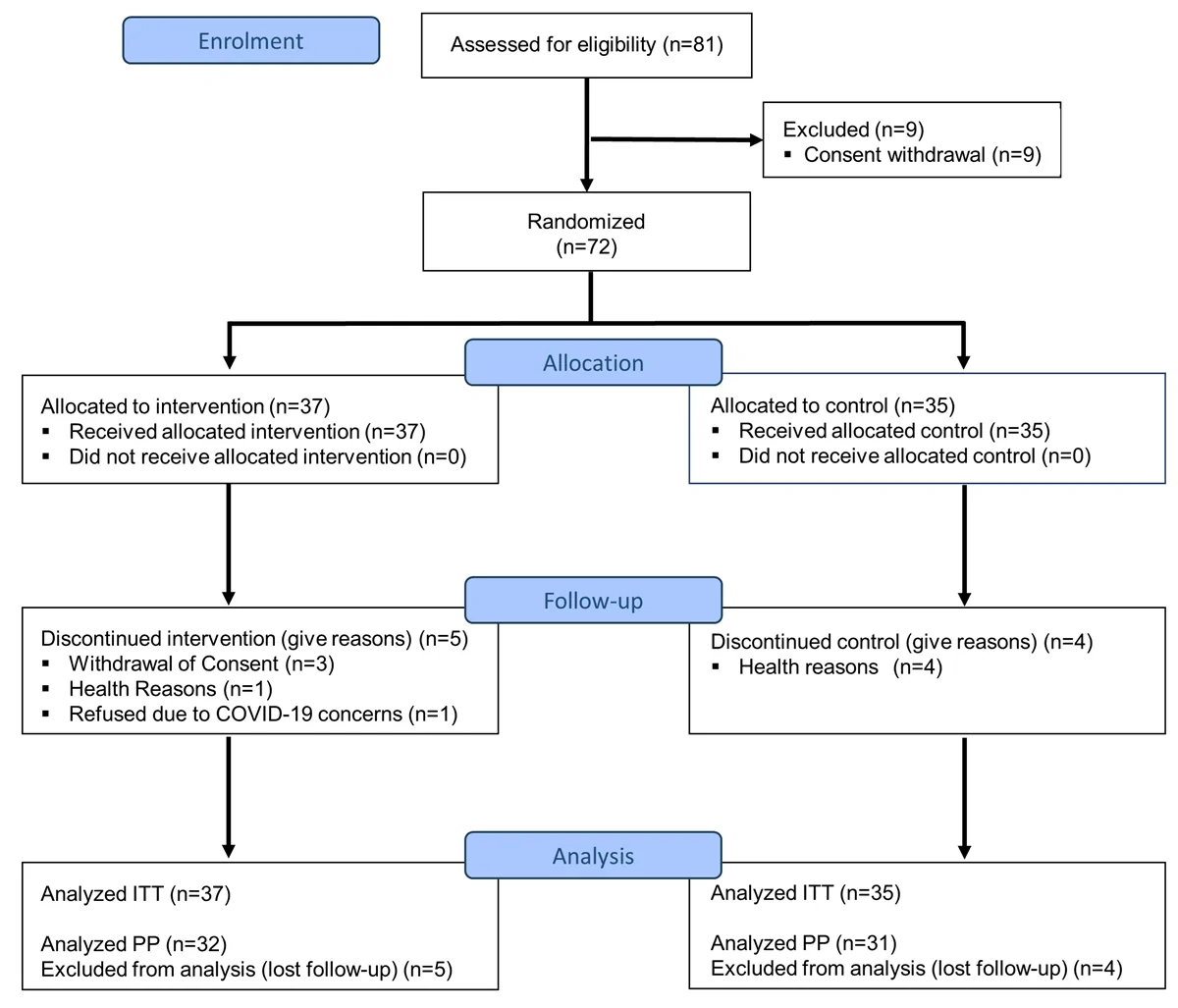
Randomized trial flow diagram. A total of 72 cognitively unimpaired older adults aged 60 years or older were randomized into the intervention (n=37) and control (n=35) groups. During the trial, 5 participants from the intervention group and 4 from the control group withdrew from the study, and 63 participants completed the 12-week follow-up assessment. Baseline and follow-up assessments included Cambridge Neuropsychological Test Automated Battery cognitive testing and brain magnetic resonance imaging acquisition. ITT: intention-to-treat; PP: per-protocol.

**Table 1:** Demographic and clinical characteristics of participants.^a^

Characteristic			Intervention (n=37)		Control (n=35)		*P* value
Demographic and clinical characteristics							
	Age (years), mean (SD)	73.49 (5.48)		73.60 (5.89)		.93	
	Education (years)^b^, mean (SD)	9.20 (4.76)		9.84 (3.93)		.53	
	Female, n (%)	31 (84)		31 (89)		.56	
	K-MMSE^c^, mean (SD)^b^	27.76 (2.24)		27.89 (1.78)		.90	
Underlying disease, n (%)							
	Diabetes mellitus^b^	10 (27)		6 (17)		.32	
	Hypertension	19 (51)		18 (51)		.99	
	Hyperlipidemia	17 (46)		13 (37)		.46	
	Dropout, n (%)^b^	5 (14)		4 (11)		.79	
Neuroimaging characteristics, mean (SD)							
	Intracranial volume (cm^3^)	1448.76 (124.66)		1464.87 (101.53)		.55	
	Whole brain volume (% of ICV^d^)	69.61 (2.55)		69.62 (2.81)		>.99	
	Global FA^e^	0.47 (0.02)		0.47 (0.02)		.22	
Baseline cognitive performance (*z* scores)^f^, mean (SD)							
	Attention	0.03 (0.92)		0.13 (0.99)		.76	
	Visuospatial	–0.22 (0.88)		–0.02 (0.76)		.08	
	Memory	0.63 (0.82)		0.9 (0.94)		.21	
	Language	0.54 (0.79)		0.32 (0.8)		.07	
	Frontal executive	0.48 (0.51)		0.57 (0.6)		.94	

^a^Data are presented as mean (SD) or number (%) for dichotomous variables. Between-group comparisons were performed using independent *t* tests unless otherwise indicated.

^b^*P* values were calculated using the Mann-Whitney *U* test for variables with nonnormal distributions.

^c^K-MMSE: Korean version of the Mini-Mental State Examination.

^d^ICV: intracranial volume.

^e^FA: fractional anisotropy.

^f^*P* values for baseline cognitive performance (*z* scores) were obtained using analysis of covariance, adjusted for age, sex, and years of education.

### Changes in WM Microstructure

At week 12, the intervention group showed significantly less reduction in FA than the control group in the right tapetum, left tapetum, and right PTR after FDR correction (*P*=.03; *P*=.03; and *P*=.04, respectively; Table 2; Figure 3). Detailed longitudinal values for each group are provided in Table S1, and within-group pre-post comparisons based on paired *t* tests are provided in Table S4 in Multimedia Appendix 2.

Regarding MD, a nominal group difference was observed in the left cingulum (cingulate gyrus; *P*=.04), but this finding did not remain significant following correction for multiple comparisons (FDR-adjusted *P*=.37; Table S5 in Multimedia Appendix 2).

In the PP analyses (participants who completed the follow-up assessment), the results were consistent with the ITT findings. Participants who adhered to the intervention protocol demonstrated less reduction in FA compared with controls at week 12 in the right tapetum (–0.03 for intervention vs –0.06 for control group; FDR-adjusted *P*=.04) and right PTR (–0.02 vs –0.03; FDR-adjusted *P*=.04; Table S6 in Multimedia Appendix 2).

**Table 2:** Changes in white matter (WM) microstructure (fractional anisotropy) measured using diffusion tensor imaging in the intention-to-treat analysis after the 12-week intervention. False discovery rate–adjusted *P* values (q) <.05 were considered statistically significant.^a^

WM microstructure			Estimated between-group difference (95% CI)		*P* value^b^		*q* ^c^
Commissural fibers							
	SCC^d^	0.010 (–0.001 to 0.020)		.07		.20	
	Tapetum (R^e^)	0.025 (0.008 to 0.043)		.004		.03	
	Tapetum (L^f^)	0.023 (0.008 to 0.039)		.003		.03	
Projection fibers							
	ACR^g^ (R)	0.003 (–0.011 to 0.017)		.66		.75	
	ACR (L)	0.001 (–0.008 to 0.010)		.82		.83	
	PTR^h^ (R)	0.013 (0.003 to 0.022)		.007		.04	
	PTR (L)	0.008 (–0.001 to 0.016)		.07		.20	
Association fibers							
	SS^i^ (R)	0.012 (–0.001 to 0.025)		.07		.20	
	SS (L)	0.005 (–0.004 to 0.013)		.27		.46	
	Cingulum-CG^j^ (R)	0.003 (–0.009 to 0.015)		.67		.75	
	Cingulum-CG (L)	0.009 (–0.004 to 0.022)		.20		.37	
	Cingulum-HPC^k^ (R)	0.013 (–0.017 to 0.042)		.40		.56	
	Cingulum-HPC (L)	0.018 (–0.009 to 0.044)		.19		.37	
	SLF^l^ (R)	0.004 (–0.004 to 0.012)		.31		.47	
	SLF (L)	0.004 (–0.002 to 0.011)		.18		.37	
	UF^m^ (R)	0.004 (–0.012 to 0.020)		.60		.75	
	UF (L)	0.002 (–0.014 to 0.017)		.83		.83	

^a^Fractional anisotropy values are adjusted for age, sex, years of education, and baseline diffusion tensor imaging measures.

^b^Uncorrected *P* value from linear mixed models.

^c^False discovery rate–adjusted *P* value using the Benjamini-Hochberg method.

^d^SCC: splenium of the corpus callosum.

^e^R: right.

^f^L: left.

^g^ACR: anterior corona radiata.

^h^PTR: posterior thalamic radiation.

^i^SS: sagittal stratum.

^j^CG: cingulum (cingulate gyrus).

^k^HPC: cingulum (hippocampus).

^l^SLF: superior longitudinal fasciculus.

^m^UF: uncinate fasciculus.

**Figure 3:**
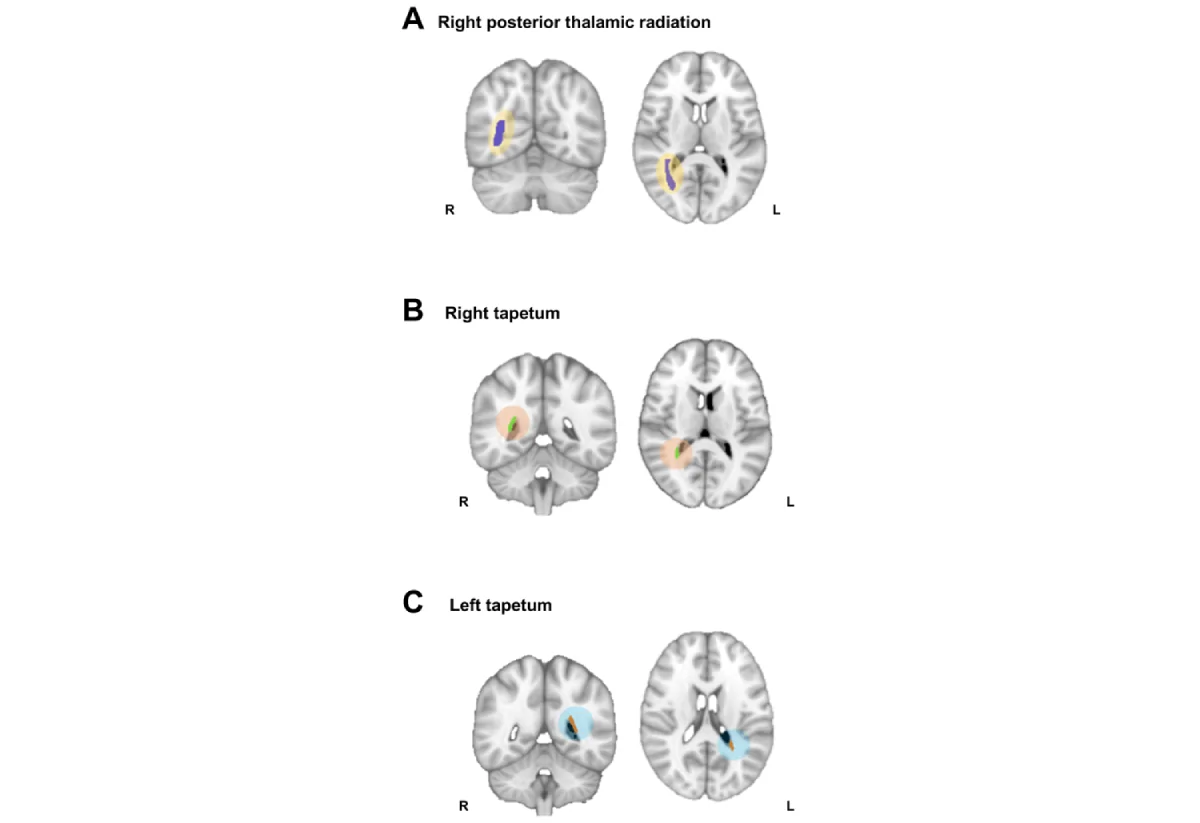
Clusters showing between-group differences in longitudinal changes in fractional anisotropy. Projection and commissural fibers are shown: (A) right posterior thalamic radiation, (B) right tapetum, and (C) left tapetum. Colored regions indicate atlas-defined region of interest locations used for region of interest–based analyses.

### Cognitive Outcomes

#### Episodic Memory (PAL)

In the ITT analysis, a linear mixed-effects model showed a significant between-group difference for PAL (estimated between-group difference 0.375, 95% CI 0.017 to 0.733; *P*=.04; Table 3 and Figure 4). Detailed longitudinal values for each group, including baseline, week 12, and within-group mean changes, are provided in Table S2 in Multimedia Appendix 2. A similar pattern for PAL was observed in the PP analysis (*P*=.049; Table S7 in Multimedia Appendix 2).

**Table 3:** Changes in cognitive outcomes measured by z scores after the 12-week intervention.^a^

Cognitive performance		Estimated between-group difference (95% CI)	*P* value
Memory			
	Delayed Matching to Sample	0.164 (–0.421 to 0.748)	.58
	Paired Associates Learning	0.375 (0.017 to 0.733)	.04
Attention			
	Rapid Visual Information Processing	–0.723 (–3.006 to 1.561)	.54
Processing speed			
	Reaction Time	–0.000 (–0.493 to 0.493)	>.99
Executive function			
	Spatial Working Memory	–0.223 (–0.697 to 0.251)	.36
	One Touch Stockings of Cambridge	0.014 (–0.464 to 0.492)	.96

^a^Adjusted for age, sex, years of education, and baseline Cambridge Neuropsychological Test Automated Battery scores. For all listed tasks, scores were transformed into *z* scores and, where necessary, oriented so that higher values indicate better cognitive performance. *P* values were not corrected for multiple comparisons across cognitive outcomes. Paired Associates Learning was the main cognitive measure of episodic memory; delayed matching to sample, Rapid Visual Information Processing, Reaction Time, Spatial Working Memory, and One Touch Stockings of Cambridge were additional cognitive outcomes.

**Figure 4:**
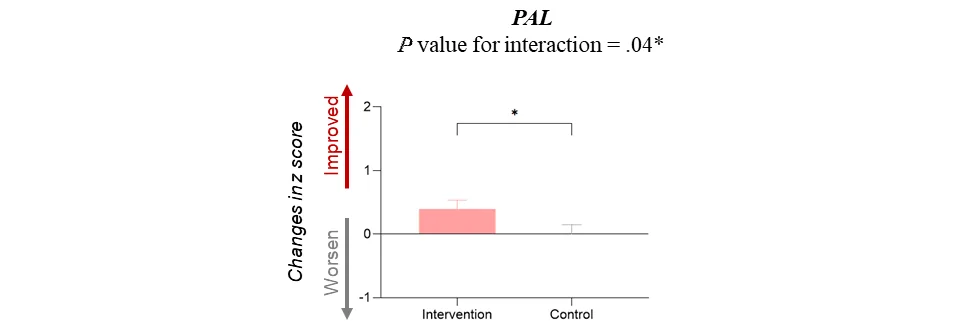
Effects of the intervention on cognitive outcomes. Models were adjusted for age, sex, years of education, and baseline Cambridge Neuropsychological Test Automated Battery scores. PAL: Paired Associates Learning.

#### Other Cognitive Functions

No significant group × time interactions were observed for other cognitive domains including DMS (*P*=.58), RVP (*P*=.54), RTI (*P*>.99), SWM (*P*=.36), or OTS (*P*=.96; Table 3 and Figure S1 in Multimedia Appendix 2). These null results were consistent across both ITT and PP analyses. Thus, cognitive improvement was observed for PAL, whereas no detectable intervention-related changes were found in the other cognitive outcomes over the 12-week intervention period.

#### Structure-Function Correlations

In the ITT analysis, significant positive associations were observed between changes in FA and improvement in PAL performance in the right PTR and the right SS, and both remained significant after FDR correction (*P*=.04 for each; Table 4 and Figure 5). No other ROI demonstrated significant associations after correction for multiple comparisons. For MD, a nominal correlation was identified in the left ACR in the ITT analysis (*P*=.01), but this association did not survive FDR correction (Table S8 in Multimedia Appendix 2). In the PP analysis, nominal correlations were observed for FA in the right PTR (*P*=.03), right SS (*P*=.03), and right SLF (*P*=.03), and for MD in the right ACR (*P*=.02) and left ACR (*P*=.004); however, no additional ROI remained significant after multiple-comparison correction (Table S9 in Multimedia Appendix 2).

**Table 4:** Partial correlations between changes in Paired Associates Learning (PAL) performance and longitudinal changes in white matter microstructure (fractional anisotropy).^a^

Anatomical region		PAL			
		*r*	*P* value^b^	*P* value^c^	*q* ^d^
Commissural					
	SCC^e^	0.167	.23	>.99	.39
	Tapetum (R^f^)	0.179	.19	>.99	.38
	Tapetum (L^g^)	0.064	.64	>.99	.72
Projection					
	ACR^h^ (R)	0.219	.10	>.99	.29
	ACR (L)	0.301	.03	.53	.18
	PTR^i^ (R)	0.417	.003	.048	.04
	PTR (L)	0.080	.60	>.99	.72
Association					
	SS^j^ (R)	0.364	.004	.08	.04
	SS (L)	0.162	.28	>.99	.39
	Cingulum-CG^k^ (R)	0.158	.27	>.99	.39
	Cingulum-CG (L)	0.204	.18	>.99	.38
	Cingulum-HPC^l^ (R)	0.180	.20	>.99	.38
	Cingulum-HPC (L)	0.032	.80	>.99	.80
	SLF^m^ (R)	0.234	.09	>.99	.29
	SLF (L)	0.252	.07	>.99	.29
	UF^n^ (R)	0.054	.71	>.99	.76
	UF (L)	–0.149	.30	>.99	.39

^a^All cognitive performance scores were oriented such that higher values indicate better performance; therefore, positive changes in PAL *z* scores reflect improvements in cognitive performance. Analyses were adjusted for age, sex, years of education, baseline cognitive scores, and diffusion tensor imaging metric at baseline. Change scores were calculated as follow-up minus baseline values.

^b^Uncorrected *P* value from partial correlation analysis.

^c^Bonferroni-corrected *P* value.

^d^False discovery rate–adjusted *P* value using the Benjamini-Hochberg method.

^e^SCC: splenium of the corpus callosum.

^f^R: right.

^g^L: left.

^h^ACR: anterior corona radiata.

^i^PTR: posterior thalamic radiation.

^j^SS: sagittal stratum.

^k^CG: cingulum (cingulate gyrus).

^l^HPC: cingulum (hippocampus).

^m^SLF: superior longitudinal fasciculus.

^n^UF: uncinate fasciculus.

**Figure 5:**
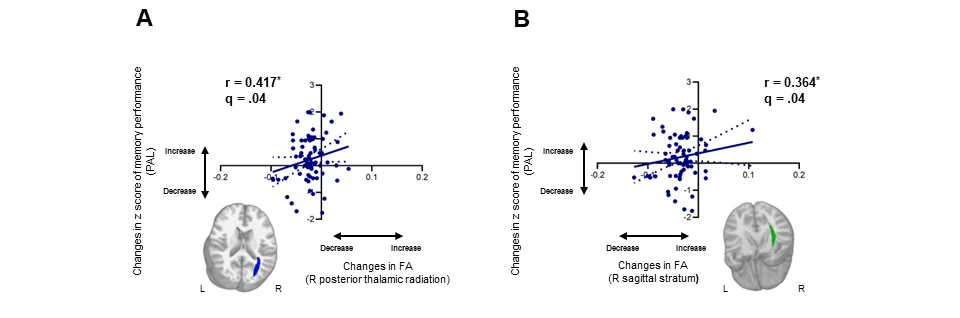
Associations between longitudinal changes in fractional anisotropy (FA) and episodic memory performance (PAL). (A) Right (R) posterior thalamic radiation. (B) Right sagittal stratum. Scatterplots show changes from baseline to week 12 in FA and PAL z scores. Solid lines indicate fitted linear relationships. Reported r values denote correlation coefficients, and q values denote FDR-adjusted *P* values. Insets show the anatomical locations of each tract. Analyses were adjusted for age, sex, years of education, and baseline imaging and cognitive scores. *q<.05.

### Intervention Use and Exploratory Dose-Response Analysis

Participants used the program for a mean of 30.73 (SD 12.84) minutes per active day and were active on a mean of 64.24 (SD 16.65) days over the 12-week intervention period, corresponding to approximately 5.35 (SD 1.39) active days per week. Participants completed a mean of 372.52 (SD 200.44) in-app training sessions. The mean total training time was 33.62 (SD 18.53) hours, and the mean time-based adherence rate was 112.1% (SD 61.8%), indicating that participants, on average, exceeded the prescribed minimum training dose of 30 total hours across 12 weeks. In unadjusted exploratory association analyses within the intervention group, the number of active days was positively correlated with PAL *z* score improvement (*r*=.374; *P*=.04), suggesting that more frequent engagement with the intervention was associated with greater memory improvement. No significant associations were observed between app-use metrics and FA changes.

## Discussion

### Principal Findings

This study demonstrated that a 12-week chatbot-based cognitive training intervention was associated with attenuated decline in FA within selected WM tracts and improved episodic memory in CU older adults, a pattern broadly consistent with a brain maintenance framework.

One of the major findings of this study was that DTI analysis revealed attenuated decline in FA in the intervention group compared with controls, most notably in the right PTR and the bilateral tapetum. Notably, significant group-by-time effects were observed primarily in FA, whereas complementary diffusivity metrics (MD, AD, and RD) showed largely null findings after multiple-comparison correction. FA is a composite measure influenced by fiber coherence, myelination, and axonal properties; thus, the present results are best interpreted as relative maintenance of microstructural organization rather than definitive structural restoration. Given the 12-week intervention duration and the modest effect sizes typically observed in diffusion metrics, detectable changes in MD/RD/AD may require longer follow-up and/or larger samples. These tracts are major long-range projection and commissural pathways that support interregional communication and have been implicated in cognitive aging [22,52]. While WM changes in older adults are often widespread, selective differences in projection and commissural fibers observed here are consistent with previous work suggesting activity-related modulation of myelinated pathways [53].

Projection fibers such as the PTR support posterior thalamo-cortical integration, which is relevant to visuospatial information processing and broader cognitive functions [52]. Given that PAL involves visuospatial associative binding [32,34], the observed attenuated FA decline in the right PTR may reflect relative maintenance of long-range pathways supporting visuospatial associative memory. Similarly, commissural fibers such as the tapetum showed attenuated FA decline, reinforcing their role in interhemispheric communication and integration. These commissural fibers support efficient interhemispheric information transfer, which is relevant to memory and higher-order cognition—functions that are particularly vulnerable to age-related decline [54-56].

Furthermore, the intervention group showed significant improvement in PAL performance, a task particularly sensitive to episodic memory. This domain‑specific response aligns with the design of the Saemi program, in which memory training was a core, recurring element. The selective improvement in memory is consistent with previous studies reporting task‑specific benefits of targeted cognitive interventions [8]. Because PAL is a visuospatial associative memory task, the observed improvement may reflect both a memory-related benefit and alignment between the intervention content and the specific cognitive demands of the task. Accordingly, these findings should not be interpreted as evidence of broad cognitive transfer across multiple domains. In contrast, no significant intervention-related changes were observed in the other cognitive outcomes, including attention, processing speed, executive function, and visuospatial recognition memory. This pattern suggests that the intervention effect on cognition was selective to episodic memory rather than generalized across all measured cognitive domains. The nonsignificant findings in the other cognitive outcomes may reflect the memory-focused structure of the intervention, limited transfer to less directly trained domains, measurement sensitivity, the relatively short intervention duration, or limited statistical power to detect smaller effects.

These findings are more consistent with brain maintenance reflected by attenuated microstructural decline than with newly induced structural restoration. Because diffusion metrics provide an indirect index of WM microstructure and do not directly quantify myelin or synapse formation, these findings should not be interpreted as evidence of structural “restoration.” Nevertheless, repeated engagement in cognitively demanding tasks has been linked to experience-dependent neuroplastic processes—including myelin-related changes and synaptic remodeling—in prior work [27,57,58], which posits that relative maintenance of brain integrity supports sustained cognitive function in older adults [21]. Although these findings are conceptually aligned with a brain-maintenance framework, the absence of longer-term follow-up assessments precludes definitive conclusions regarding the durability of this mechanism. In summary, the observed pattern is more consistent with relative maintenance of WM microstructural organization in pathways relevant to cognitive integration and memory processing, which may be related to intervention-associated improvements in episodic memory [46,53] and is conceptually aligned with a brain maintenance framework [21].

Building on the observed intervention effects on both cognitive performance and WM microstructure, we explored whether changes in these domains were interrelated. Using the imputed ITT dataset, exploratory partial correlation analyses were performed across all participants to examine whether changes in PAL performance were associated with changes in WM microstructure. Change scores were calculated as follow-up minus baseline values. Positive PAL *z* score changes indicate improvement in performance, whereas positive FA changes indicate relative preservation or increase in FA. Partial correlation analyses suggested an association between changes in episodic memory performance and WM microstructural integrity in task-relevant pathways. In addition, PAL improvement was also associated with FA change in the right SS, an associative pathway that includes major posterior cortico-cortical fibers (eg, inferior fronto-occipital and inferior longitudinal components) supporting visual information transfer and integration. Given that PAL relies on visuospatial associative binding, the concurrent PTR-SS associations may suggest that relative maintenance of posterior projection and associative pathways is related to memory-related performance during aging. However, these exploratory structure-function correlations cannot establish causal or mechanistic relationships and should be interpreted as hypothesis-generating findings that require confirmation in larger studies with longer-term follow-up. Nevertheless, this pattern is broadly consistent with the brain maintenance framework, which emphasizes relative preservation of neural structure and function in successful cognitive aging [21,22,52].

Taken together, these findings suggest a possible structure-function relationship in which improvements in episodic memory were associated with relative maintenance of white matter microstructural organization, consistent with a brain maintenance interpretation. Rather than demonstrating a definitive mechanism, our findings indicate that better episodic memory performance was associated with relatively preserved WM pathways implicated in memory-related processing. This interpretation is consistent with the integrated maintenance model, which emphasizes the contribution of both cognitive and structural resilience to successful cognitive aging [21,53,59,60].

### Limitations and Future Directions

Several limitations should be acknowledged. First, the relatively small sample size may have limited the statistical power to detect smaller effect sizes and reduced the generalizability of the findings. Future studies with larger, more diverse samples are needed to confirm these results. Second, DTI-derived outcomes were not listed as outcomes in the trial registration, although DTI was included in the IRB-approved neuroimaging protocol. Therefore, these findings should be interpreted as a focused analysis rather than as the primary confirmatory findings of the registered trial. Third, although previous studies suggest that cognitive training effects can persist long-term [1,61], this study assessed only the immediate postintervention effects of a 12-week program. Therefore, the durability and longitudinal trajectory of the observed cognitive and WM changes could not be determined. In addition, without follow-up assessments beyond the intervention period, it remains unclear whether the observed WM differences reflect sustained neuroplastic change or more transient fluctuations. Longitudinal follow-up studies with repeated assessments over time are necessary to clarify the durability of these effects and the potential need for booster sessions. Fourth, potentially influential lifestyle factors such as sleep, physical activity, and social engagement were not assessed. These factors are known to affect WM integrity and may interact with cognitive training effects. Future research could consider multimodal interventions that integrate cognitive, behavioral, and lifestyle components [62]. Fifth, although the intervention emphasized memory training, likely contributing to the significant PAL improvements, no effects were observed in other cognitive domains. This domain-specific pattern suggests that the cognitive benefit was not broadly generalized across domains. Because PAL is a visuospatial associative memory task, the observed improvement may reflect both a memory-related benefit and alignment between the training content and the specific task demands. Future iterations of the program could adjust the training balance or incorporate more targeted modules to evaluate broader transfer effects. Another limitation is that the study did not include an active control condition. Accordingly, nonspecific effects such as expectancy, engagement, general mental stimulation, or digital interaction cannot be fully excluded. Therefore, the observed effects should be interpreted cautiously and should not be attributed solely to the specific cognitive-training content of the intervention. Future studies should include an active control condition matched for contact time, digital interaction, and engagement to better distinguish the specific effects of chatbot-based cognitive training from nonspecific intervention-related effects.

Nonetheless, this study demonstrated that a 12-week chatbot-based cognitive intervention was associated with improved episodic memory and attenuated decline in WM microstructural integrity within key neural pathways. These findings provide early evidence that chatbot-based cognitive training was associated with improvements in episodic memory and attenuated decline in WM microstructural integrity, suggesting potential cognitive and neurostructural effects that warrant confirmation in larger trials.

## Appendix

Multimedia Appendix 1 CONSORT-eHEALTH checklist (V 1.6.1).

Multimedia Appendix 2 Supplementary tables and figures.

## Abbreviations

ACR: anterior corona radiata
AD: axial diffusivity
CANTAB: Cambridge Neuropsychological Test Automated Battery
CONSORT-EHEALTH: Consolidated Standards of Reporting Trials of Electronic and Mobile Health Applications and Online Telehealth
CRIS: Clinical Research Information Service
CU: cognitively unimpaired
DMS: delayed matching to sample
DTI: diffusion tensor imaging
FA: fractional anisotropy
FDR: false discovery rate
FLIRT: FMRIB’s Linear Image Registration Tool
fMRI: functional magnetic resonance imaging
FMRIB: Functional Magnetic Resonance Imaging of the Brain
FSL: FMRIB Software Library
IRB: institutional review board
ITT: intention-to-treat
MD: mean diffusivity
MRI: magnetic resonance imaging
OTS: One Touch Stockings of Cambridge
PAL: Paired Associates Learning
PP: per-protocol
PTR: posterior thalamic radiation
RD: radial diffusivity
ROI: region of interest
RTI: Reaction Time
RVP: Rapid Visual Information Processing
SHAP: Shapley additive explanations
SLF: superior longitudinal fasciculus
SNSB-II: Seoul Neuropsychological Screening Battery, second edition
SS: sagittal stratum
SWM: Spatial Working Memory
TBSS: tract-based spatial statistics
TE: echo time
TR: repetition time
WM: white matter

## Data Availability

Deidentified participant data (including the data dictionary) and statistical code are available from the corresponding author upon reasonable request, subject to institutional and ethical approvals.
