# Supplementary material for: Impact of Chatbot-Based Cognitive Training on Cognitive Function and White Matter Integrity in Cognitively Unimpaired Older Adults: Randomized Controlled Trial
Source: JMIR Aging. 2026 Oct 28;9:e94127. doi: 10.2196/94127 (PMC13755777; doi:10.2196/94127)
Supplement: Supplementary file 2 [file aging_v9i1e94127_app2.docx]

**Table S1.** Primary outcome: Changes in WM microstructure (FA) measured using DTI in the intention-to-treat analysis after the 12-week Intervention.

| **WM microstructure** | |  | **Intervention** | | | |  |  |  | **Control** | | | |  |  |  | **Intervention vs control** |  | ***P*** | ***P*ᵇ** | **q** |
| --- | --- | --- | --- | --- | --- | --- | --- | --- | --- | --- | --- | --- | --- | --- | --- | --- | --- | --- | --- | --- | --- |
|  |  |  | **(N = 37)** | | | |  |  |  | **(N = 35)** | | | |  |  |  |  |  |  |  |  |
|  |  |  | **Baseline** | | **Week 12** | |  | **Mean change (95% CI)** |  | **Baseline** | | **Week 12** | |  | **Mean change (95% CI)** |  | **Estimated between-group difference**  **(95% CI)** |  |  |  |  |
|  |  |  | *mean* | *SD* | *mean* | *SD* |  |  |  | *mean* | *SD* | *mean* | *SD* |  |  |  |  |  |  |  |  |
| *Commissural fibers* | | | | | | |  |  |  |  |  |  |  |  |  |  |  |  |  |  |  |
|  | SCC |  | 0.81 | 0.02 | 0.77 | 0.03 |  | -0.04  (-0.04 to -0.03) |  | 0.80 | 0.02 | 0.75 | 0.02 |  | -0.05  (-0.05 to -0.04) |  | 0.010 (-0.001 to 0.020) |  | 0.067 | 1 | 0.198 |
| *R* | Tapetum |  | 0.58 | 0.06 | 0.55 | 0.06 |  | -0.03  (-0.04 to -0.00) |  | 0.57 | 0.07 | 0.52 | 0.07 |  | -0.05  (-0.06 to -0.04) |  | 0.025 (0.008 to 0.043) |  | 0.004 | 0.069 | **0.034^*^** |
| L | Tapetum |  | 0.61 | 0.06 | 0.60 | 0.06 |  | -0.01  (-0.03 to -0.00) |  | 0.57 | 0.07 | 0.52 | 0.07 |  | -0.05  (-0.05 to -0.03) |  | 0.023 (0.008 to 0.039) |  | 0.003 | 0.055 | **0.034^*^** |
| Projection fibers | | | | | | |  |  |  |  |  |  |  |  |  |  |  |  |  |  |  |
| R | ACR |  | 0.46 | 0.04 | 0.45 | 0.04 |  | -0.01  (-0.02 to 0.00) |  | 0.45 | 0.03 | 0.43 | 0.04 |  | -0.02  (-0.02 to -0.01) |  | 0.003 (-0.011 to 0.017) |  | 0.663 | 1 | 0.754 |
| L | ACR |  | 0.45 | 0.04 | 0.43 | 0.03 |  | -0.02  (-0.03 to -0.01) |  | 0.44 | 0.03 | 0.42 | 0.03 |  | -0.02  (-0.02 to -0.01) |  | 0.001 (-0.008 to 0.010) |  | 0.824 | 1 | 0.832 |
| *R* | PTR |  | 0.6 | 0.04 | 0.58 | 0.03 |  | -0.02  (-0.03 to -0.00) |  | 0.59 | 0.03 | 0.56 | 0.03 |  | -0.03  (-0.04 to -0.02) |  | 0.013 (0.003 to 0.022) |  | 0.007 | 0.119 | **0.040^*^** |
| L | PTR |  | 0.59 | 0.04 | 0.58 | 0.03 |  | -0.01  (-0.02 to -0.00) |  | 0.59 | 0.03 | 0.56 | 0.04 |  | -0.03  (-0.03 to -0.01) |  | 0.008 (-0.001 to 0.016) |  | 0.070 | 1 | 0.198 |
| Association fibers | | | | | | | |  |  |  |  |  |  |  |  |  |  |  |  |  |  |
| R | SS |  | 0.55 | 0.04 | 0.52 | 0.03 |  | -0.03  (-0.04 to -0.02) |  | 0.54 | 0.02 | 0.50 | 0.02 |  | -0.04  (-0.05 to -0.03) |  | 0.012 (-0.001 to 0.025) |  | 0.066 | 1 | 0.198 |
| L | SS |  | 0.54 | 0.04 | 0.5 | 0.03 |  | -0.04  (-0.04 to -0.02) |  | 0.53 | 0.02 | 0.49 | 0.03 |  | -0.04  (-0.04 to -0.03) |  | 0.005 (-0.004 to 0.013) |  | 0.270 | 1 | 0.459 |
| R | Cingulum–CG |  | 0.6 | 0.03 | 0.53 | 0.04 |  | -0.07  (-0.07 to -0.05) |  | 0.58 | 0.04 | 0.51 | 0.04 |  | -0.07  (-0.07 to -0.06) |  | 0.003 (-0.009 to 0.015) |  | 0.665 | 1 | 0.754 |
| L | Cingulum–CG |  | 0.63 | 0.04 | 0.57 | 0.04 |  | -0.06  (-0.07 to -0.05) |  | 0.61 | 0.04 | 0.54 | 0.04 |  | -0.07  (-0.08 to -0.06) |  | 0.009 (-0.004 to 0.022) |  | 0.195 | 1 | 0.368 |
| R | Cingulum–HPC |  | 0.59 | 0.06 | 0.5 | 0.08 |  | -0.09  (-0.11 to -0.06) |  | 0.57 | 0.05 | 0.47 | 0.05 |  | -0.11  (-0.11 to -0.08) |  | 0.013 (-0.017 to 0.042) |  | 0.396 | 1 | 0.561 |
| L | Cingulum–HPC |  | 0.55 | 0.04 | 0.51 | 0.07 |  | -0.04  (-0.06 to -0.02) |  | 0.55 | 0.05 | 0.48 | 0.05 |  | -0.07  (-0.08 to -0.05) |  | 0.018 (-0.009 to 0.044) |  | 0.187 | 1 | 0.368 |
| R | SLF |  | 0.53 | 0.03 | 0.51 | 0.03 |  | -0.02  (-0.02 to -0.01) |  | 0.52 | 0.03 | 0.50 | 0.02 |  | -0.02  (-0.02 to -0.02) |  | 0.004 (-0.004 to 0.012) |  | 0.306 | 1 | 0.473 |
| L | SLF |  | 0.54 | 0.03 | 0.51 | 0.03 |  | -0.03  (-0.03 to -0.02) |  | 0.53 | 0.02 | 0.49 | 0.02 |  | -0.04  (-0.03 to -0.03) |  | 0.004 (-0.002 to 0.011) |  | 0.177 | 1 | 0.368 |
| R | UF |  | 0.58 | 0.05 | 0.55 | 0.05 |  | -0.03  (-0.04 to -0.01) |  | 0.57 | 0.04 | 0.53 | 0.04 |  | -0.04  (-0.04 to -0.02) |  | 0.004 (-0.012 to 0.020) |  | 0.600 | 1 | 0.754 |
| L | UF |  | 0.51 | 0.05 | 0.5 | 0.04 |  | -0.01  (-0.02 to 0.01) |  | 0.51 | 0.05 | 0.50 | 0.05 |  | -0.01  (-0.03 to 0.00) |  | 0.002 (-0.014 to 0.017) |  | 0.832 | 1 | 0.832 |

Fractional anisotropy (FA) values are adjusted for age, sex, years of education, and baseline DTI measures.

P: uncorrected P value from linear mixed models (LMM); Pᵇ: Bonferroni-corrected P value; q: false discovery rate (FDR)–adjusted P value using the Benjamini–Hochberg method.

*q<.05, **q<.01, ***q<.001

Abbreviations: ACR, anterior corona radiata; CG, cingulum (cingulate gyrus); FA, fractional anisotropy; HPC, cingulum (hippocampus); L, left; PAL, paired associates learning; PTR, posterior thalamic radiation; R, right; SCC, splenium of the corpus callosum; SLF, superior longitudinal fasciculus; SS, sagittal stratum; UF, uncinate fasciculus.

**Table S2.** Changes in secondary cognitive outcomes measured by z-scores using intention-to-treat after 12-week intervention.

| **Cognitive performance** |  | **Intervention** | | | |  |  |  | **Control** | | | |  |  |  | **Intervention vs control** |  | ***P*** |
| --- | --- | --- | --- | --- | --- | --- | --- | --- | --- | --- | --- | --- | --- | --- | --- | --- | --- | --- |
|  |  | **(N = 37)** | | | |  |  |  | **(N = 35)** | | | |  |  |  |  |  |  |
|  |  | **Baseline** | | **Week 12** | |  | **Mean change  (95% CI)** |  | **Baseline** | | **Week 12** | |  | **Mean change  (95% CI)** |  | **Estimated between-group difference (95% CI)** |  |  |
|  |  | *mean* | *SD* | *mean* | *SD* |  |  |  | *mean* | *SD* | *mean* | *SD* |  |  |  |  |  |  |
| **Memory** | | | |  |  |  |  |  |  |  |  |  |  |  |  |  |  |  |
| Delayed Matching to Sample (DMS) |  | -0.16 | 1.04 | -0.16 | 1.13 |  | 0  (-0.391 to 0.425) |  | -0.00 | 1.00 | -0.14 | 1.32 |  | -0.14  (-0.564 to 0.270) |  | 0.164  (-0.421 to 0.748) |  | 0.583 |
| Paired Associates Learning (PAL) |  | 0.01 | 1.00 | 0.47 | 0.90 |  | 0.46  (0.155 to 0.655) |  | 0.00 | 1.00 | 0.00 | 1.10 |  | 0  (-0.226 to 0.285) |  | 0.375  (0.017 to 0.733) |  | **0.040^*^** |
| **Attention** |  |  |  |  |  |  |  |  |  |  |  |  |  |  |  |  |  |  |
| Rapid Visual Information Processing (RVP) |  | 0.12 | 0.86 | 0.32 | 0.96 |  | 0.20  (-0.040 to 0.462) |  | 0.00 | 1.00 | 0.10 | 0.80 |  | 0.10  (-0.112 to 0.402) |  | -0.723  (-3.006 to 1.561) |  | 0.535 |
| **Processing speed** |  |  |  |  |  |  |  |  |  |  |  |  |  |  |  |  |  |  |
| Reaction Time (RTI) |  | 0.19 | 0.99 | 0.46 | 0.93 |  | 0.27  (-0.072 to 0.617) |  | 0.00 | 1.00 | 0.24 | 1.31 |  | 0.24  (-0.080 to 0.624) |  | -0.000  (-0.493 to 0.493) |  | 0.999 |
| **Executive function** | | |  |  |  |  |  |  |  |  |  |  |  |  |  |  |  |  |
| Spatial Working Memory (SWM) |  | -0.48 | 1.44 | -0.10 | 1.09 |  | 0.38  (0.062 to 0.725) |  | 0.00 | 1.00 | 0.20 | 0.88 |  | 0.20  (-0.168 to 0.509) |  | -0.223  (-0.697 to 0.251) |  | 0.357 |
| One Touch Stockings of Cambridge (OTS) |  | 0.32 | 0.88 | -0.41 | 1.03 |  | -0.73  (-1.109 to -0.442) |  | 0.00 | 1.00 | -0.83 | 0.97 |  | -0.83  (-1.130 to -0.448) |  | 0.014  (-0.464 to 0.492) |  | 0.955 |

Adjusted for age, sex, years of education and baseline CANTAB scores, ^*^P<.05.

Abbreviation: DMS, Delayed Matching to Sample; OTS, One Touch Stockings of Cambridge; PAL, paired associates learning; RTI, reaction time; RVP, Rapid Visual Information Processing; SD, standard deviation; SWM, Spatial Working Memory.

**Table S3.** Seventeen predefined regions of interest (ROIs) with label numbers from the JHU-ICBM DTI-81 white-matter atlas

| Label number | Parcellation | Label name |
| --- | --- | --- |
|  |  |  |
| 5 | Commissural | splenium of the corpus callosum |
| 47 | Commissural | tapetum R |
| 48 | Commissural | tapetum L |
| 23 | Projection | anterior corona radiata R |
| 24 | Projection | anterior corona radiata L |
| 29 | Projection | posterior thalamic radiation (including optic radiation) R |
| 30 | Projection | posterior thalamic radiation (including optic radiation) L |
| 31 | Association | sagittal stratum R |
| 32 | Association | sagittal stratum L |
| 35 | Association | cingulum (cingulate gyrus) R |
| 36 | Association | cingulum (cingulate gyrus) L |
| 37 | Association | cingulum (hippocampus) R |
| 38 | Association | cingulum (hippocampus) L |
| 41 | Association | superior longitudinal fasciculus R |
| 42 | Association | superior longitudinal fasciculus L |
| 45 | Association | uncinate fasciculus R |
| 46 | Association | uncinate fasciculus L |

Abbreviations: L, left; R, right;

**Table S4.** Comparison of WM integrity pre- and post-intervention using paired t-test.

| **Anatomical Region** | | | **Baseline** | | **Follow up** | |  | **Mean change  (95% CI)** | | |  | **t-statistic** | ***P*** |
| --- | --- | --- | --- | --- | --- | --- | --- | --- | --- | --- | --- | --- | --- |
|  |  |  | **mean** | **SD** | **mean** | **SD** |  |  |  |  |  |  |  |
| *Commissural fibers* | |  |  |  |  |  |  |  |  |  |  |  |  |
|  | SCC |  | 0.81 | 0.02 | 0.77 | 0.03 |  | -0.04 (-0.04 -0.03) | | |  | -14.06 | <.01^**^ |
| R | Tapetum |  | 0.57 | 0.06 | 0.54 | 0.06 |  | -0.04 (-0.05 -0.03) | | |  | -6.72 | <.01^**^ |
| L | Tapetum |  | 0.61 | 0.06 | 0.59 | 0.05 |  | -0.03 (-0.04 -0.02) | | |  | -5.92 | <.01^**^ |
| *Projection fibers* | |  |  |  |  |  |  |  | | |  |  |  |
| R | ACR |  | 0.45 | 0.04 | 0.44 | 0.04 |  | -0.01 (-0.02 -0.01) | | |  | -3.40 | <.01^**^ |
| L | ACR |  | 0.44 | 0.03 | 0.43 | 0.03 |  | -0.01 (-0.02 -0.01) | | |  | -5.55 | <.01^**^ |
| R | PTR |  | 0.60 | 0.03 | 0.57 | 0.03 |  | -0.03 (-0.03 -0.02) | | |  | -6.78 | <.01^**^ |
| L | PTR |  | 0.59 | 0.03 | 0.58 | 0.03 |  | -0.01 (-0.02 -0.01) | | |  | -5.55 | <.01^**^ |
| *Association fibers* | |  |  |  |  |  |  |  | | |  |  |  |
| R | SS |  | 0.55 | 0.03 | 0.51 | 0.03 |  | -0.04 (-0.04 -0.03) | | |  | -8.93 | <.01^**^ |
| L | SS |  | 0.53 | 0.03 | 0.50 | 0.03 |  | -0.03 (-0.04 -0.03) | | |  | -11.20 | <.01^**^ |
| R | Cingulum–CG |  | 0.59 | 0.04 | 0.53 | 0.04 |  | -0.06 (-0.07 -0.06) | | |  | -19.69 | <.01^**^ |
| L | Cingulum–CG |  | 0.62 | 0.04 | 0.56 | 0.04 |  | -0.06 (-0.07 -0.06) | | |  | -16.63 | <.01^**^ |
| R | Cingulum–HPC |  | 0.58 | 0.06 | 0.49 | 0.06 |  | -0.09 (-0.11 -0.07) | | |  | -11.16 | <.01^**^ |
| L | Cingulum–HPC |  | 0.55 | 0.05 | 0.50 | 0.06 |  | -0.05 (-0.07 -0.04) | | |  | -7.86 | <.01^**^ |
| R | SLF |  | 0.57 | 0.05 | 0.55 | 0.05 |  | -0.02 (-0.02 -0.01) | | |  | -8.36 | <.01^**^ |
| L | SLF |  | 0.51 | 0.05 | 0.50 | 0.04 |  | -0.01 (-0.03 -0.02) | | |  | -15.24 | <.01^**^ |
| R | UF |  | 0.57 | 0.06 | 0.54 | 0.06 |  | -0.03 (-0.04 -0.02) | | |  | -6.27 | <.01^**^ |
| L | UF |  | 0.61 | 0.06 | 0.59 | 0.05 |  | -0.02 (-0.02 0.00) | | |  | -2.02 | <.01^**^ |

^*^*P*<.05, ^**^*P*<.01

Abbreviations: ACR, anterior corona radiata; CG, cingulum (cingulate gyrus); HPC, cingulum (hippocampus); L, left; PTR, posterior thalamic radiation; R, right; SCC, splenium of the corpus callosum; SLF, superior longitudinal fasciculus; SS, sagittal stratum; UF, uncinate fasciculus.

**Table S5.** Primary outcome: Changes in WM microstructure (**MD)** measured using DTI in the intention-to-treat analysis after 12-week Intervention.

| **WM microstructure** | |  | **Intervention** | | | |  |  |  | **Control** | | | |  |  |  | **Intervention vs control** |  | ***P*** | ***P*ᵇ** | **q** |
| --- | --- | --- | --- | --- | --- | --- | --- | --- | --- | --- | --- | --- | --- | --- | --- | --- | --- | --- | --- | --- | --- |
|  |  |  | **(N = 37)** | | | |  |  |  | **(N = 35)** | | | |  |  |  |  |  |  |  |  |
|  | |  | **Baseline** | | **Week 12** | |  | **Mean change (95% CI)** |  | **Baseline** | | **Week 12** | |  | **Mean change (95% CI)** |  | **Estimated between-group difference (95% CI)** |  |  |  |  |
|  |  |  | *mean* | *SD* | *mean* | *SD* |  |  |  | *mean* | *SD* | *mean* | *SD* |  |  |  |  |  |  |  |  |
| *Commissural fibers* | | | |  |  |  |  |  |  |  |  |  |  |  |  |  |  |  |  |  |  |
|  | SCC |  | 0.00070 | 0.00003 | 0.00086 | 0.00018 |  | 0.00016 (0.00010 to 0.00023) |  | 0.00071 | 0.00003 | 0.00085 | 0.00010 |  | 0.00014 (0.00011 to 0.00018) |  | 0.00002 (-0.00004 to 0.00009) |  | 0.529 | 1 | 0.692 |
| R | Tapetum |  | 0.00091 | 0.00009 | 0.00106 | 0.00041 |  | 0.00015 (0.00001 to 0.00030) |  | 0.00092 | 0.00009 | 0.00097 | 0.00024 |  | 0.00004 (-0.00006 to 0.00013) |  | 0.00011 (-0.00004 to 0.00026) |  | 0.151 | 1 | 0.374 |
| L | Tapetum |  | 0.0009 | 0.00011 | 0.00102 | 0.00041 |  | 0.00018 (0.00003 to 0.00033) |  | 0.0009 | 0.00010 | 0.0009 | 0.0002 |  | 0.00006 (-0.00003 to 0.00016) |  | 0.00010 (-0.00005 to 0.00025) |  | 0.186 | 1 | 0.395 |
| *Projection fibers* | | | |  |  |  |  |  |  |  |  |  |  |  |  |  |  |  |  |  |  |
| R | ACR |  | 0.00077 | 0.00005 | 0.00078 | 0.00007 |  | 0.00000 (-0.00002 to 0.00002) |  | 0.00079 | 0.00005 | 0.00082 | 0.00008 |  | 0.00002 (0.00000 to 0.00004) |  | -0.00002 (-0.00004 to 0.00001) |  | 0.138 | 1 | 0.374 |
| L | ACR |  | 0.00078 | 0.00005 | 0.00081 | 0.00007 |  | 0.00003 (0.00000 to 0.00005) |  | 0.00079 | 0.00004 | 0.00083 | 0.00008 |  | 0.00003 (0.00001 to 0.00006) |  | -0.00001 (-0.00003 to 0.00002) |  | 0.597 | 1 | 0.725 |
| R | PTR |  | 0.00080 | 0.00005 | 0.00085 | 0.00016 |  | 0.00005 (-0.00001 to 0.00011) |  | 0.00081 | 0.00004 | 0.00088 | 0.00015 |  | 0.00006 (0.00000 to 0.00012) |  | -0.00001 (-0.00008 to 0.00006) |  | 0.692 | 1 | 0.735 |
| L | PTR |  | 0.00083 | 0.00005 | 0.00091 | 0.00019 |  | 0.00007 (-0.00000 to 0.00014) |  | 0.00085 | 0.00005 | 0.00088 | 0.00013 |  | 0.00003 (-0.00002 to 0.00008) |  | 0.00004 (-0.00003 to 0.00012) |  | 0.288 | 1 | 0.487 |
| *Association fibers* | | | |  |  |  |  |  |  |  |  |  |  |  |  |  |  |  |  |  |  |
| R | SS |  | 0.00078 | 0.00005 | 0.00088 | 0.00013 |  | 0.00010 (0.00005 to 0.00014) |  | 0.00080 | 0.00004 | 0.00092 | 0.00010 |  | 0.00012 (0.00008 to 0.00016) |  | -0.00002 (-0.00007 to 0.00003) |  | 0.441 | 1 | 0.625 |
| L | SS |  | 0.00083 | 0.00005 | 0.00093 | 0.00013 |  | 0.00010 (0.00006 to 0.00014) |  | 0.00083 | 0.00005 | 0.00098 | 0.00014 |  | 0.00015 (0.00010 to 0.00020) |  | -0.00004 (-0.00010 to 0.00001) |  | 0.146 | 1 | 0.374 |
| R | Cingulum–CG |  | 0.00069 | 0.00003 | 0.00087 | 0.00018 |  | 0.00018 (0.00011 to 0.00024) |  | 0.00070 | 0.00004 | 0.00093 | 0.00013 |  | 0.00022 (0.00018 to 0.00027) |  | -0.00004 (-0.00012 to 0.00003) |  | 0.211 | 1 | 0.399 |
| L | Cingulum–CG |  | 0.00069 | 0.00004 | 0.00087 | 0.00018 |  | 0.00018 (0.00012 to 0.00025) |  | 0.00070 | 0.00003 | 0.00097 | 0.00017 |  | 0.00027 (0.00021 to 0.00033) |  | -0.00008 (-0.00016 to -0.00000) |  | 0.040 | 0.680 | 0.374 |
| R | Cingulum–HPC |  | 0.00067 | 0.00006 | 0.00100 | 0.00039 |  | 0.00033 (0.00018 to 0.00047) |  | 0.00070 | 0.00006 | 0.00118 | 0.00039 |  | 0.00047 (0.00032 to 0.00062) |  | -0.00013 (-0.00031 to 0.00005) |  | 0.153 | 1 | 0.374 |
| L | Cingulum–HPC |  | 0.00074 | 0.00005 | 0.00101 | 0.00030 |  | 0.00027 (0.00016 to 0.00038) |  | 0.00075 | 0.00008 | 0.00110 | 0.00032 |  | 0.00035 (0.00022 to 0.00047) |  | -0.00007 (-0.00021 to 0.00007) |  | 0.315 | 1 | 0.487 |
| R | SLF |  | 0.00070 | 0.00003 | 0.00077 | 0.00008 |  | 0.00007 (0.00005 to 0.00010) |  | 0.00071 | 0.00003 | 0.00082 | 0.00010 |  | 0.00010 (0.00007 to 0.00014) |  | -0.00003 (-0.00007 to 0.00001) |  | 0.141 | 1 | 0.374 |
| L | SLF |  | 0.00071 | 0.00003 | 0.00079 | 0.00009 |  | 0.00008 (0.00005 to 0.00011) |  | 0.00072 | 0.00003 | 0.00083 | 0.00009 |  | 0.00011 (0.00008 to 0.00014) |  | -0.00003 (-0.00007 to 0.00001) |  | 0.154 | 1 | 0.374 |
| R | UF |  | 0.00069 | 0.00004 | 0.00081 | 0.00014 |  | 0.00012 (0.00006 to 0.00017) |  | 0.00071 | 0.00004 | 0.00083 | 0.00008 |  | 0.00012 (0.00009 to 0.00015) |  | -0.00000 (-0.00006 to 0.00005) |  | 0.868 | 1 | 0.868 |
| L | UF |  | 0.00079 | 0.00008 | 0.00093 | 0.00027 |  | 0.00013 (0.00003 to 0.00023) |  | 0.00079 | 0.00007 | 0.00091 | 0.00011 |  | 0.00011 (0.00006 to 0.00015) |  | 0.00002 (-0.00007 to 0.00012) |  | 0.657 | 1 | 0.735 |

Mean diffusivity (MD) values are adjusted for age, sex, years of education, and baseline DTI measures.

P: uncorrected P value from linear mixed models (LMM); Pᵇ: Bonferroni-corrected P value; q: false discovery rate (FDR)–adjusted P value using the Benjamini–Hochberg method.

Abbreviations: ACR, anterior corona radiata; CG, cingulum (cingulate gyrus); HPC, cingulum (hippocampus); L, left; MD, mean diffusivity; PTR, posterior thalamic radiation; R, right; SCC, splenium of the corpus callosum; SLF, superior longitudinal fasciculus; SS, sagittal stratum; UF, uncinate fasciculus.

**Table S6.** Primary outcome: Changes in WM microstructure (FA) measured using DTI in the **Per-protocol** analysis after 12-week Intervention.

| **WM microstructure** | |  | **Intervention** | | | |  |  |  | **Control** | | | |  |  |  | **Intervention vs control** | |  | ***P*** | ***P*ᵇ** | **q** |
| --- | --- | --- | --- | --- | --- | --- | --- | --- | --- | --- | --- | --- | --- | --- | --- | --- | --- | --- | --- | --- | --- | --- |
|  |  |  | **(N = 37)** | | | |  |  |  | **(N = 35)** | | | |  |  |  |  |  |  |  |  |  |
|  | |  | **Baseline** | | **Week 12** | |  | **Mean change (95% CI)** |  | **Baseline** | | **Week 12** | |  | **Mean change (95% CI)** |  | **Estimated between-group difference (95% CI)** |  | |  |  |  |
|  |  |  | *mean* | *SD* | *mean* | *SD* |  |  |  | *mean* | *SD* | *mean* | *SD* |  |  |  |  |  |  |  |  |  |
| Commissural fibers | | |  |  |  |  |  |  |  |  |  |  |  |  |  |  |  |  | |  |  |  |
|  | SCC |  | 0.81 | 0.02 | 0.77 | 0.03 |  | -0.036 (-0.044 to -0.027) |  | 0.80 | 0.02 | 0.75 | 0.02 |  | -0.046 (-0.054 to -0.037) |  | 0.010 (-0.001 to 0.021) |  | | 0.065 | 1 | 0.221 |
| R | Tapetum |  | 0.58 | 0.05 | 0.55 | 0.06 |  | -0.028 (-0.045 to -0.011) |  | 0.57 | 0.07 | 0.52 | 0.07 |  | -0.055 (-0.067 to -0.042) |  | 0.027 (0.008 to 0.045) |  | | 0.005 | 0.082 | **0.043^*^** |
| L | Tapetum |  | 0.62 | 0.05 | 0.60 | 0.06 |  | -0.022 (-0.036 to -0.009) |  | 0.61 | 0.06 | 0.57 | 0.05 |  | -0.044 (-0.056 to -0.032) |  | 0.022 (0.005 to 0.038) |  | | 0.009 | 0.160 | 0.051 |
| Projection fibers | | |  |  |  |  |  |  |  |  |  |  |  |  |  |  |  |  | |  |  |  |
| R | ACR |  | 0.46 | 0.03 | 0.45 | 0.04 |  | -0.012 (-0.026 to 0.002) |  | 0.45 | 0.03 | 0.43 | 0.04 |  | -0.016 (-0.023 to -0.008) |  | 0.004 (-0.011 to 0.019) |  | | 0.636 | 1 | 0.768 |
| L | ACR |  | 0.44 | 0.04 | 0.43 | 0.03 |  | -0.015 (-0.024 to -0.005) |  | 0.44 | 0.03 | 0.42 | 0.03 |  | -0.016 (-0.022 to -0.010) |  | 0.001 (-0.009 to 0.011) |  | | 0.806 | 1 | 0.856 |
| R | PTR |  | 0.60 | 0.03 | 0.58 | 0.03 |  | -0.018 (-0.026 to -0.010) |  | 0.59 | 0.03 | 0.56 | 0.03 |  | -0.032 (-0.040 to -0.023) |  | 0.014 (0.004 to 0.024) |  | | 0.004 | 0.074 | **0.043^*^** |
| L | PTR |  | 0.59 | 0.03 | 0.58 | 0.03 |  | -0.011 (-0.018 to -0.005) |  | 0.58 | 0.03 | 0.56 | 0.04 |  | -0.019 (-0.026 to -0.012) |  | 0.008 (-0.001 to 0.017) |  | | 0.081 | 1 | 0.230 |
| Association fibers | | |  |  |  |  |  |  |  |  |  |  |  |  |  |  |  |  | |  |  |  |
| R | SS |  | 0.55 | 0.03 | 0.52 | 0.03 |  | -0.030 (-0.044 to -0.016) |  | 0.54 | 0.03 | 0.50 | 0.02 |  | -0.044 (-0.052 to -0.035) |  | 0.014 (-0.000 to 0.027) |  | | 0.051 | 0.87 | 0.217 |
| L | SS |  | 0.54 | 0.03 | 0.50 | 0.03 |  | -0.033 (-0.039 to -0.027) |  | 0.53 | 0.02 | 0.49 | 0.03 |  | -0.038 (-0.046 to -0.030) |  | 0.005 (-0.004 to 0.014) |  | | 0.258 | 1 | 0.439 |
| R | Cingulum–CG |  | 0.60 | 0.03 | 0.53 | 0.04 |  | -0.062 (-0.073 to -0.051) |  | 0.58 | 0.04 | 0.51 | 0.04 |  | -0.065 (-0.073 to -0.057) |  | 0.003 (-0.010 to 0.015) |  | | 0.678 | 1 | 0.768 |
| L | Cingulum–CG |  | 0.63 | 0.04 | 0.57 | 0.04 |  | -0.060 (-0.071 to -0.049) |  | 0.61 | 0.04 | 0.54 | 0.04 |  | -0.069 (-0.080 to -0.058) |  | 0.009 (-0.005 to 0.023) |  | | 0.207 | 1 | 0.439 |
| R | Cingulum–HPC |  | 0.59 | 0.06 | 0.50 | 0.08 |  | -0.087 (-0.117 to -0.058) |  | 0.57 | 0.06 | 0.47 | 0.05 |  | -0.100 (-0.119 to -0.082) |  | 0.013 (-0.018 to 0.044) |  | | 0.416 | 1 | 0.589 |
| L | Cingulum–HPC |  | 0.56 | 0.04 | 0.51 | 0.07 |  | -0.048 (-0.072 to -0.025) |  | 0.54 | 0.05 | 0.48 | 0.05 |  | -0.065 (-0.084 to -0.046) |  | 0.017 (-0.011 to 0.044) |  | | 0.236 | 1 | 0.439 |
| R | SLF |  | 0.53 | 0.03 | 0.51 | 0.03 |  | -0.017 (-0.025 to -0.009) |  | 0.52 | 0.03 | 0.50 | 0.02 |  | -0.021 (-0.026 to -0.017) |  | 0.004 (-0.004 to 0.013) |  | | 0.285 | 1 | 0.440 |
| L | SLF |  | 0.53 | 0.03 | 0.51 | 0.03 |  | -0.026 (-0.031 to -0.020) |  | 0.52 | 0.02 | 0.49 | 0.02 |  | -0.031 (-0.035 to -0.026) |  | 0.005 (-0.002 to 0.011) |  | | 0.149 | 1 | 0.362 |
| R | UF |  | 0.58 | 0.05 | 0.55 | 0.05 |  | -0.027 (-0.041 to -0.013) |  | 0.57 | 0.04 | 0.53 | 0.04 |  | -0.032 (-0.044 to -0.020) |  | 0.005 (-0.012 to 0.022) |  | | 0.572 | 1 | 0.748 |
| L | UF |  | 0.51 | 0.05 | 0.50 | 0.04 |  | -0.005 (-0.019 to 0.009) |  | 0.50 | 0.05 | 0.50 | 0.05 |  | -0.005 (-0.019 to 0.008) |  | 0.000 (-0.017 to 0.017) |  | | 0.985 | 1 | 0.985 |

Fractional anisotropy (FA) values are adjusted for age, sex, years of education, and baseline DTI measures.

P: uncorrected P value from linear mixed models (LMM); Pᵇ: Bonferroni-corrected P value; q: false discovery rate (FDR)–adjusted P value using the Benjamini–Hochberg method.

^*^q<.05, ^**^q<.01, ^***^ q<.001.

Abbreviations: ACR, anterior corona radiata; CG, cingulum (cingulate gyrus); FA, fractional anisotropy; HPC, cingulum (hippocampus); L, left; MD, mean diffusivity; PTR, posterior thalamic radiation; R, right; SCC, splenium of the corpus callosum; SLF, superior longitudinal fasciculus; SS, sagittal stratum; UF, uncinate fasciculus.

**Table S7.** Changes in cognitive outcomes measured by cognitive test z-scores in the per-protocol analysis after the 12-week intervention.

| **Cognitive performance** |  | **Intervention** | | | |  |  |  | **Control** | | | |  |  |  | **Intervention vs control** |  | ***P*** |
| --- | --- | --- | --- | --- | --- | --- | --- | --- | --- | --- | --- | --- | --- | --- | --- | --- | --- | --- |
|  |  |  | | | |  |  |  |  | | | |  |  |  |  |  |  |
|  |  | **Baseline** | | **Week 12** | |  | **Mean change**  **(95% CI)** |  | **Baseline** | | **Week 12** | |  | **Mean change (95% CI)** |  | **Estimated between-group difference (95% CI)** |  |  |
|  |  | *mean* | *SD* | *mean* | *SD* |  |  |  | *mean* | *SD* | *mean* | *SD* |  |  |  |  |  |  |
| **Memory** |  |  |  |  |  |  |  |  |  |  |  |  |  |  |  |  |  |  |
| Delayed Matching to Sample (DMS) |  | -0.19 | 0.92 | -0.16 | 1.13 |  | 0.033 (-0.463 to 0.529) |  | 0.03 | 1.04 | -0.14 | 1.32 |  | -0.170 (-0.669 to 0.330) |  | 0.203 (-0.414 to 0.819) |  | 0.519 |
| Paired Associates Learning (PAL) |  | 0.07 | 0.99 | 0.47 | 0.90 |  | 0.401 (0.102 to 0.700) |  | -0.02 | 0.98 | 0.00 | 1.10 |  | 0.021 (-0.286 to 0.327) |  | 0.381 (0.001 to 0.760) |  | 0.049^*^ |
| **Attention** |  |  |  |  |  |  |  |  |  |  |  |  |  |  |  |  |  |  |
| Rapid Visual Information Processing (RVP) |  | 0.34 | 1.71 | 1.57 | 4.26 |  | 1.226 (-0.359 to 2.811) |  | 0.04 | 1.06 | 1.98 | 5.78 |  | 1.935 (-0.168 to 4.038) |  | -0.709 (-3.140 to 1.722) |  | 0.568 |
| **Processing speed** |  |  |  |  |  |  |  |  |  |  |  |  |  |  |  |  |  |  |
| Reaction Time (RTI) |  | 0.19 | 1.06 | 0.46 | 0.93 |  | 0.271 (-0.114 to 0.655) |  | -0.06 | 1.01 | 0.24 | 1.31 |  | 0.294 (-0.163 to 0.752) |  | -0.024 (-0.547 to 0.500) |  | 0.930 |
| **Executive function** |  |  |  |  |  |  |  |  |  |  |  |  |  |  |  |  |  |  |
| Spatial Working Memory (SWM) |  | -0.50 | 1.48 | -0.10 | 1.09 |  | 0.395 (-0.006 to 0.796) |  | 0.03 | 1.01 | 0.20 | 0.88 |  | 0.163 (-0.291 to 0.617) |  | 0.232 (-0.270 to 0.734) |  | 0.365 |
| One Touch Stockings of Cambridge (OTS) |  | 0.36 | 0.88 | -0.41 | 1.03 |  | -0.771 (-1.260 to -0.281) |  | -0.02 | 0.99 | -0.83 | 0.97 |  | -0.812 (-1.196 to -0.428) |  | 0.041 (-0.465 to 0.548) |  | 0.873 |

Data are presented as the mean (standard deviation). Adjusted for age, sex, years of education and baseline CANTAB scores.

**P*<.05.

Abbreviation: DMS, Delayed Matching to Sample; OTS, One Touch Stockings of Cambridge; PAL, paired associates learning; RTI, reaction time; RVP, Rapid Visual Information Processing; SD, standard deviation; SWM, Spatial Working Memory

**Table S8.** Exploratory partial correlations between changes in cognitive function z-scores and longitudinal changes in white matter microstructure (MD) in the imputed analysis dataset.

| **Anatomical Region** | | |  | **PAL** | | | |
| --- | --- | --- | --- | --- | --- | --- | --- |
|  |  |  |  | **r** | ***P*** | ***P*ᵇ** | **q** |
| ***MD*** |  |  |  |  |  |  |  |
| *Commissural fibers* |  | SCC |  | 0.038 | 0.771 | 1 | 0.981 |
|  | R | Tapetum |  | -0.058 | 0.658 | 1 | 0.981 |
|  | L | Tapetum |  | -0.056 | 0.665 | 1 | 0.981 |
| *Projection fibers* | R | ACR |  | -0.217 | 0.103 | 1 | 0.879 |
|  | L | ACR |  | -0.308 | 0.014 | 0.234 | 0.234 |
|  | R | PTR |  | 0.033 | 0.808 | 1 | 0.981 |
|  | L | PTR |  | 0.046 | 0.724 | 1 | 0.981 |
| *Association fibers* | R | SS |  | 0.081 | 0.543 | 1 | 0.981 |
|  | L | SS |  | -0.052 | 0.696 | 1 | 0.981 |
|  | R | Cingulum–CG |  | -0.067 | 0.599 | 1 | 0.981 |
|  | L | Cingulum–CG |  | -0.002 | 0.989 | 1 | 0.989 |
|  | R | Cingulum–HPC |  | 0.010 | 0.938 | 1 | 0.989 |
|  | L | Cingulum–HPC |  | 0.004 | 0.977 | 1 | 0.989 |
|  | R | SLF |  | -0.158 | 0.213 | 1 | 0.981 |
|  | L | SLF |  | -0.090 | 0.492 | 1 | 0.981 |
|  | R | UF |  | 0.066 | 0.625 | 1 | 0.981 |
|  | L | UF |  | 0.080 | 0.536 | 1 | 0.981 |

P: uncorrected P value from partial correlation analysis; Pᵇ: Bonferroni-corrected P value; q: false discovery rate (FDR)–adjusted P value using the Benjamini–Hochberg method.

Analyses adjusted for age, sex, years of education, baseline cognitive scores, and DTI metric at baseline.

^*^q<.05, ^**^q<.01, ^***^ q<.001.

Abbreviations: ACR, anterior corona radiata; CG, cingulum (cingulate gyrus); HPC, cingulum (hippocampus); L, left; MD, mean diffusivity; PAL, paired associates learning; PTR, posterior thalamic radiation; R, right; SCC, splenium of the corpus callosum; SLF, superior longitudinal fasciculus; SS, sagittal stratum; UF, uncinate fasciculus.

**Table S9.** Exploratory partial correlations between changes in cognitive function z-scores and longitudinal changes in white matter microstructure (FA and MD) among participants who completed the 12-week follow-up assessment.

| **Anatomical Region** | | |  |  | **PAL** |  |  |  |  | **PAL** |  |  |
| --- | --- | --- | --- | --- | --- | --- | --- | --- | --- | --- | --- | --- |
|  |  |  |  | **r** | ***P*** | ***P*ᵇ** | **q** |  | **r** | ***P*** | ***P*ᵇ** | **q** |
|  |  |  |  | ***FA*** |  |  |  |  | ***MD*** |  |  |  |
| Commissural fibers |  | SCC |  | 0.066 | 0.623 | 1 | 0.756 |  | 0.148 | 0.269 | 1 | 0.733 |
|  | R | Tapetum |  | 0.22 | 0.098 | 1 | 0.325 |  | -0.142 | 0.287 | 1 | 0.733 |
|  | L | Tapetum |  | 0.105 | 0.435 | 1 | 0.725 |  | -0.081 | 0.543 | 1 | 0.733 |
| Projection fibers | R | ACR |  | 0.097 | 0.469 | 1 | 0.725 |  | -0.305 | 0.020 | 0.339 | 0.17 |
|  | L | ACR |  | 0.209 | 0.115 | 1 | 0.325 |  | -0.373 | 0.004 | 0.066 | 0.066 |
|  | R | PTR |  | 0.29 | 0.027 | 0.462 | 0.191 |  | 0.098 | 0.465 | 1 | 0.733 |
|  | L | PTR |  | 0.053 | 0.695 | 1 | 0.787 |  | -0.028 | 0.837 | 1 | 0.874 |
| Association fibers | R | SS |  | 0.287 | 0.029 | 0.492 | 0.191 |  | 0.139 | 0.297 | 1 | 0.733 |
|  | L | SS |  | 0.134 | 0.317 | 1 | 0.6 |  | 0.023 | 0.866 | 1 | 0.874 |
|  | R | Cingulum–CG |  | -0.081 | 0.544 | 1 | 0.756 |  | 0.021 | 0.874 | 1 | 0.874 |
|  | L | Cingulum–CG |  | 0.025 | 0.855 | 1 | 0.876 |  | 0.147 | 0.272 | 1 | 0.733 |
|  | R | Cingulum–HPC |  | 0.152 | 0.254 | 1 | 0.541 |  | 0.04 | 0.768 | 1 | 0.874 |
|  | L | Cingulum–HPC |  | 0.021 | 0.876 | 1 | 0.876 |  | 0.088 | 0.512 | 1 | 0.733 |
|  | R | SLF |  | 0.279 | 0.034 | 0.574 | 0.191 |  | -0.121 | 0.367 | 1 | 0.733 |
|  | L | SLF |  | 0.219 | 0.099 | 1 | 0.325 |  | 0.132 | 0.324 | 1 | 0.733 |
|  | R | UF |  | 0.075 | 0.578 | 1 | 0.756 |  | 0.088 | 0.513 | 1 | 0.733 |
|  | L | UF |  | -0.197 | 0.139 | 1 | 0.337 |  | 0.078 | 0.560 | 1 | 0.733 |

Fractional anisotropy (FA) and Mean diffusivity (MD) values are presented. Results were adjusted for age, sex, years of education, and baseline DTI measures. Uncorrected P values, Bonferroni-corrected P values (Pᵇ), and FDR-adjusted q values are reported.

^*^q<.05, ^**^q<.01, ^***^ q<.001.

Abbreviations: ACR, anterior corona radiata; CG, cingulum (cingulate gyrus); FA, fractional anisotropy; HPC, cingulum (hippocampus); L, left; MD, mean diffusivity; PAL, paired associates learning; PTR, posterior thalamic radiation; R, right; SCC, splenium of the corpus callosum; SLF, superior longitudinal fasciculus; SS, sagittal stratum; UF, uncinate fasciculus.

**Supplementary Figure S1**. Secondary cognitive outcomes by cognitive domain.

**Supplementary Figure S2**. SHAP summary plot for atlas-defined white matter ROIs associated with PAL change.
